# On the Tincture of Iodine in Frost-Bite

**Published:** 1841-03-20

**Authors:** D. M. Wright

**Affiliations:** Edenton, N. C.


### MEDICAL EXAMINER.

DEVOTED TO MEDICINE, SURGERY, AND THE COLLATERAL SCIENCES.

No. 12.] PHILADELPHIA, SATURDAY, MARCH 20, 1841. [Vol. IV.

### ORIGINAL COMMUNICATIONS.

*On the Tincture of Iodine in Frost-Bite.* By D. M. Wright, M. D.

To the Editors of the Medical Examiner.

On looking over the pages of one of the late numbers of the American Medical Library, I was struck with the singular efficacy which was ascribed to the use of the tinct. of iodine in erysipelas, burns, furuncle, chilblains, and other varieties of inflammation; and coming from so respectable a source as it did, I determined to give it a trial where an opportunity should permit. A few days after I was consulted by a couple of seamen, who, having been exposed in the recent very severe weather, were suffering greatly from frost-bite. The hands of one of them were much swollen and rincated, the ends of the fingers quite insensible, and a feeling of numbness in the whole hand. A painful prickling sensation was produced by pressure, which also vibrated; great quickness in the capillary circulation. The pulse at the ends was remarkably full and strong; but this activity seemed confined to the wrists; the patient did not have general fever. The other did not suffer so violently; his hands, however, were much swollen, and quite painful. I directed their hands to be dipped in hot water, and a strong tincture of iodine brushed over the inflamed part twice a day. A few applications were sufficient to relieve entirely the one who suffered least severely; and the other, by persevering in the treatment about a week, is now quite relieved. His hands pealed all over; and the nails from three of his fingers are detached; a new skin formed above as readily as the old pealed off, and his hands are now as fair and delicate as a lady's, his fingers perfectly flexible, quite sensitive, but without soreness, and the nails are beginning to form in the fingers from which they were detached; no other treatment has been resorted to in the case. I have seen but few cases of frost-bite, and none so severe as the above described, and have ever found it very tedious and difficult of cure; I am convinced had I been acquainted with the efficacy of the above named remedy, I should have had much less difficulty. I would recommend it to the notice of your readers, and am convinced that one trial of it will cause them to discard all other remedies in such cases.

I am satisfied that iodine is not fully appreciated as a remedial agent, and I was much pleased to see its claims as an internal remedy set forth in a recent number of your paper. I think it is destined shortly to become a very useful and popular remedy. I am informed by my friend, Dr. Charles E. Johnson, a practitioner in a neighbouring village, and a gentleman in whose judgment and veracity all reliance may be placed, that he is in the habit of using it frequently in combination with sulph. quinine in cases of intermittents in which there are visceral engorgements, and which have resisted the ordinary methods of treatment, and that the practice has been unusually successful. He thinks the combination is admissible in many cases in which the quinine separately would not be borne, and that its administration may often supersede the necessity of resorting to mercurials as alteratives. The tincture was of the strength recommended in the dispensatory of Wood and Bache.

*Edenton, N. C.*, March 8th, 1841.

